# FAT1 mutation-related signature predicts survival risk and tumor immunogenicity in lung adenocarcinoma

**DOI:** 10.3389/fgene.2025.1466484

**Published:** 2025-07-02

**Authors:** Lifeng Gao, Xueying Wang, Yixin Xu, Aimin Wang, Wenjing Zhang, Qinghua Wang, Yanfeng Ren

**Affiliations:** ^1^ Department of Clinical Laboratory, Affiliated Hospital of Shandong Second Medical University, Weifang, Shandong, China; ^2^ Key Laboratory of Medicine and Health of Shandong Province, Department of Health Statistics, School of Public Health, Shandong Second Medical University, Weifang, Shandong, China; ^3^ School of Medical Laboratory, Shandong Second Medical University, Weifang, Shandong, China

**Keywords:** lung adenocarcinoma, FAT1 mutation signature, survival risk, tumor immunogenicity, immune treatment, prognostic indicators

## Abstract

**Background:**

FAT atypical cadherin 1 (FAT1) is a well-known tumor regulator that plays a crucial role in multiple cancer signaling pathways. Its mutations have been linked to tumor progression and immune regulation in various cancers, including lung adenocarcinoma (LUAD). In this study, we aim to identify a FAT1 mutation-related transcriptomic risk signature to assess the survival risks and immune status of LUAD patients.

**Methods:**

A total of 2528 LUAD samples, which included both gene expression profiles and clinicopathologic data, were collected from 12 datasets. Additionally, two datasets treated with immunotherapies were also included to investigate the therapeutic effects.

**Results:**

We constructed a FAT1 mutation molecular signature based on 9 relevant genes. LUAD patients with low-risk scores demonstrated a more favorable prognosis compared to those with high-risk scores, which is corroborated by 6 additional independent datasets. Further immunological, mutational, and intratumor microbial analyses reveal that increased infiltration of immune effector cells, increased mutational burden, specific mutational signatures (such as age and APOBEC associated), mutations in driver genes (e.g., TP53, KEAP1, NAV3, and SMARCA4), and increased microbial α/β diversities are present in the low-risk LUAD patients. Based on the immunotherapeutic patients, an improved immune checkpoint blockade treatment prognosis and an elevated response rate are also observed in the low-risk signature group.

**Conclusion:**

In summary, Our identified FAT1 mutation-related risk signature shows potential for assessing LUAD clinical outcomes, tumor immunogenicity, and immunotherapy effectiveness, providing valuable insights for LUAD clinical practice.

## Introduction

Lung adenocarcinoma (LUAD) is a type of lung cancer that originates from cells responsible for producing mucus. This malignancy accounts for 40% of all lung cancer cases and is the most prevalent subtype (Nicholson et al., 2022). Although LUAD has a relatively favorable prognosis compared to other forms of non-small cell lung cancer (NSCLC) (Nicholson et al., 2022), there is currently a lack of effective molecular markers to accurately predict patient outcomes and disease progression. Therefore, new and effective biomarkers are urgently needed in clinical practice to evaluate the prognosis of patients with LUAD.

Immune checkpoint blockade (ICB) therapies (e.g., anti-PD-1/PD-L1 and anti-CTLA-4 treatments) use monoclonal antibodies to block inhibitory checkpoints and reactivate the immune response against cancer cells. Although ICB therapy has significantly improved the survival of cancer patients, only a small proportion of patients can benefit from it, mainly due to the lack of immunogenicity in most patients (Wang Q. et al., 2023). For example, a recent study has indicated that enhancing the activity of CD5-positive dendritic cells can activate the response of effector CD8-positive T cells to tumors, thereby improving tumor immunogenicity and further enhancing the response rate to immunotherapy (He et al., 2023).

Tumor mutation burden (TMB) reflects the enrichment of somatic mutations within the tumor genome, with a higher TMB leading to the generation of more neoantigens, subsequently enhancing T-cell recognition and improving immunotherapy response rates (Li et al., 2022). However, Goodman et al. have noted that even with high TMB levels, poor presentation of driver mutation neoantigens by MHC-I could still render tumors unresponsive to ICB therapy (Goodman et al., 2020). Shin et al. discovered that JAK1/2 loss-of-function mutations could lead to acquired or primary resistance to anti-PD-1 treatment in patients with high TMB levels (Shin et al., 2017). Furthermore, due to tumor heterogeneity, there is currently no definitive TMB cut-off value for patient stratification (Wang Y. et al., 2022; Wang Y. et al., 2023). These evidences indicate the instability of TMB in predicting the immunotherapy efficacy, suggesting that it is not a flawless clinical biomarker. High expression of PD-L1 protein is one of the predictive biomarkers for the benefit of immunotherapy in NSCLC (Wojas-Krawczyk and Kubiatowski, 2020). However, the results of the CheckMate 227 clinical trial indicated that PD-L1-negative NSCLC patients could still show a benefit from ICB treatment; additionally, a subset of patients with PD-L1-negative expression exhibited significantly prolonged survival (Hellmann et al., 2019). Furthermore, several issues remain unresolved in utilizing PD-L1 to guide immunotherapy: (1) determination of the cut-off value for PD-L1 positivity; (2) differences in results across various detection platforms; (3) variations in expression levels among different tumor types (Wojas-Krawczyk and Kubiatowski, 2020). These findings also suggest that PD-L1 expression is not a reliable marker in assessing the efficacy of tumor immunotherapy. Therefore, there is a need to further explore robust indicators for evaluating and predicting the efficacy of cancer immunotherapy.

The FAT1 gene encodes a large transmembrane protein with extracellular cadherin repeats, EGF-like domains, and laminin G-like domains that are typically expressed in epithelial tissues (Wang Z. et al., 2022). The function of FAT1 in human cancers varies depending on the type of cancer, as it can act as either an oncogene or a tumor suppressor (Chen Y. H. et al., 2022). By regulating the Hippo pathway, FAT1 influences various molecular signaling pathways such as WNT/β-catenin, TGF-β, PI3K/AKT, and others, thereby affecting tumor progression (Li et al., 2018). In breast cancer, reduced FAT1 expression was associated with high histological grade, poor lymph node status, progression, aggressive behavior, and a worse prognosis (Wang et al., 2016). Overexpression of FAT1 in NSCLC cells reduced stem cell markers and inhibited spheroid formation, potentially reducing tumor formation by promoting the nucleoplasmic translocation of YAP1 (Li et al., 2021). FAT1 mutations are common in human cancers, predominantly occurring as nonsense mutations (Pastushenko et al., 2021). A recent study conducted in mouse models of skin squamous cell carcinoma and lung cancer revealed that loss of FAT1 could accelerate tumor occurrence and malignant progression, promoting epithelial to mesenchymal transition (EMT) (Pastushenko et al., 2021). This EMT state was also observed in human squamous cell carcinomas with FAT1 mutations (Pastushenko et al., 2021). Similarly, a study based on integrated multi-omics immunotherapy cohorts demonstrated that FAT1 mutations are associated with improved immune checkpoint blockade (ICB) treatment outcomes in both non-small cell lung cancer and melanoma (Zhang et al., 2022).

Regarding the crucial role of FAT1 and its mutations in tumor progression and therapeutic prognosis, this study amalgamated 2,528 samples from 12 independent LUAD datasets to construct a molecular prognosis signature associated with FAT1 mutations. By integrating diverse omics data, including immunological characteristics, mutational features, and intratumoral microbial traits, we explored the possible molecular mechanisms underpinning the prognosis signature. The findings from this investigation are anticipated to furnish a theoretical basis for assessing prognosis and predicting treatment efficacy for LUAD.

## Materials and methods

### Acquisition of LUAD samples and corresponding multi-omics data

We collected 509 LUAD samples from the Cancer Genome Atlas (TCGA) project, including transcriptome, somatic mutation, intratumoral microbiome profiles, and clinicopathological prognosis information. These samples were used as discovery datasets to construct the FAT1 mutation-related survival risk signature. All samples included in the study must have follow-up information, both overall survival (OS) times and OS statuses. Subsequently, we verified the risk signature by collecting 11 additional LUAD datasets from the Gene Expression Omnibus (GEO) project, including GSE72094 (N = 398), GSE68465 (N = 442), GSE50081 (N = 127), GSE42127 (N = 132), GSE41271 (N = 181), GSE31210 (N = 226), GSE30219 (N = 85), GSE13213 (N = 117), GSE26939 (N = 115), GSE11969 (N = 90), and GSE81089 (N = 106). In the data processing stage, we merged samples from the same microarray platform to obtain a larger sample size and more stable results. The three datasets of GSE50081, GSE31210, and GSE30219, which share the Affymetrix Human Genome U133 Plus 2.0 Array, were combined as the pooled dataset 1; the two datasets of GSE42127 and GSE41271, which share the Illumina HumanWG-6 v3.0 expression beadchip, were combined as the pooled dataset 2. To correct for batch effects between merged datasets, we employed the ComBat function of the R sva package (Leek et al., 2012). All LUAD samples underwent chemotherapy at different stages, with a few patients undergoing immunotherapy, but detailed treatment information is not available. Supplementary Table S1 provided detailed information on all LUAD datasets included in this study and the microarray platforms.

**FIGURE 1:**
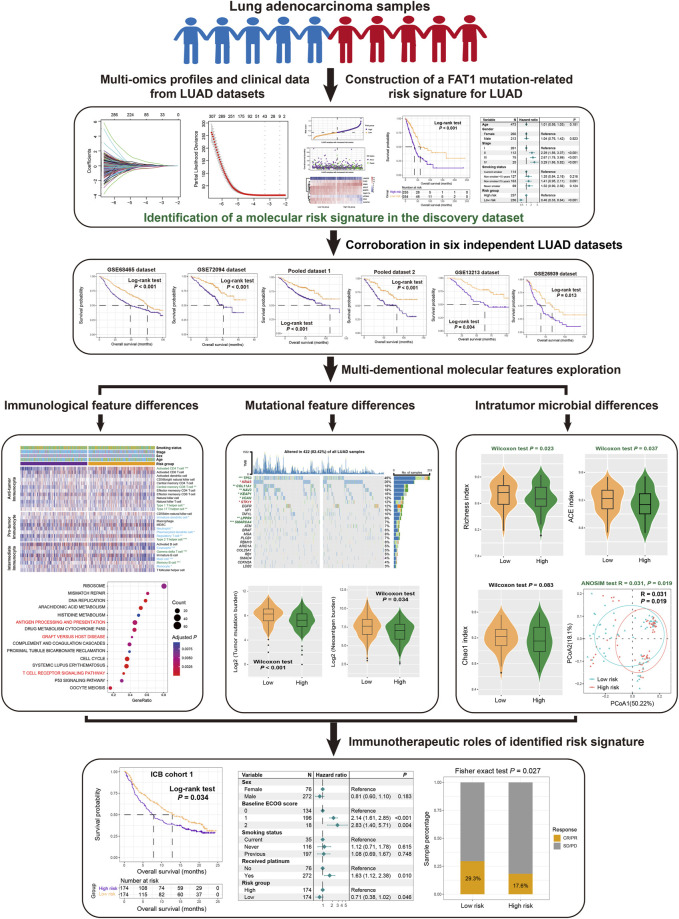
The overall design of this study is to construct a risk signature related to FAT1 mutation in LUAD patients.

To investigate the significance of FAT1 mutation risk signature in predicting therapeutic efficacy, we analyzed two cohorts comprising transcriptomic expression data and immune checkpoint blockade (ICB) treatment information. The first is IMvigor210 cohort (Mariathasan et al., 2018), contained 348 patients with advanced urothelial carcinoma who were treated with atezolizumab (anti-PD-L1 drug). Transcriptome data and treatment prognostic response information for this cohort are available at http://research-pub.gene.com/IMvigor210CoreBiologies and are referred to as ICB cohort 1. The second cohort (ICB cohort 2) consisted of 121 patients with melanoma who had received anti-PD-1/PD-L1 or combination treatments (Liu et al., 2019). Complete clinicopathologic data and immunotherapeutic information for urothelial carcinoma and melanoma patients are provided in Supplementary Tables S2, S3, respectively. The detailed design processes for this study are illustrated in Figure 1.

### Construction and corroboration of the FAT1 mutation-related risk signature

The TCGA discovery cohort was utilized to analyze transcriptome gene expression differences between FAT1 mutation and wild-type patients, all genes with *P* values less than 0.05 were determined to be associated with FAT1 mutation and were considered as potential differential markers. In the second step, univariate Cox regression analysis was performed for all FAT1 mutation-related genes to identify those that had a significant impact on prognoses. In the third step, all prognostic genes were subjected to Lasso-Cox regression analysis [implemented by R glmnet package (Friedman et al., 2010)] to select the most significant gene subsets that contributed the most to prognoses. The optimal λ was determined via 10-fold cross-validation using the 1-SE rule, which minimizes overfitting while retaining predictive power. Finally, a risk score was calculated for each LUAD patient based on the specific genes identified by Lasso regression and their corresponding regression coefficients. The detailed risk score calculation method is provided as: risk score = $\sum_{i}^{n}Coefficient\;of\;gene\;\left(i\right)*Expression\;of\;gene\;\left(i\right)$ . All patients were divided into high-risk and low-risk groups based on median scores, and the association with survival risks was analyzed. To ensure the robustness of the constructed risk signature, multiple verification datasets obtained from the GEO platform were used to validate the results. We also utilized the surv_cutpoint function within the R survminer package to stratify LUAD patients based on their signature risk scores and analyzed the survival differences between high- and low-risk subgroups across all datasets.

### Tumor infiltration immunocytes and immune checkpoints

To investigate the immune cell infiltration differences between high-risk and low-risk LUAD patients, we employed Charoentong et al. method (Charoentong et al., 2017) to provide a comprehensive estimate of 28 types of tumor infiltrating immune cells. These 28 cell subtypes were classified into three categories: anti-tumor cells, pro-tumor cells, and neutral cells. The specific gene sets used to estimate these immune cell abundances are presented in Supplementary Table S4. We also utilized the current commonly used CIBERSORT (Newman et al., 2015) and TIMER method (Li et al., 2020) to assess immune cell infiltration in LUAD patients. The CIBERSORT method evaluated a total of 22 immune cell types based on 547 feature genes in the LM22 eigenmatrix, while the TIMER method estimated the infiltration proportion of 6 major immune cells through the deconvolution operation.

Based on a previous study in the field of tumor immunogenomics (Ye et al., 2020), we collected a total of 32 immune checkpoint genes and explored the differences in their expression across different risk groups of LUAD patients.

### Tumor immunogenicity relevant signatures

Recent studies have identified a number of tumor immunogenicity-related molecular signatures that are associated with immune response and treatment sensitivity in cancer. In our study, we collected 4 representative molecular signatures for analysis: 1) T cell-inflamed signature (Ayers et al., 2017), which contains 18 genes that are involved in activating T cells and are associated with pembrolizumab therapeutic response; 2) interferon γ (IFNγ) signature (Gocher et al., 2022), a classic anti-tumor molecular signal that has been demonstrated to be related to immunogenicity and efficacy of immunotherapy; 3) cytolytic activity (Rooney et al., 2015), which indicates the ability of tumor cells to survive and is inversely correlated with their viability; and 4) WNT TGF-β signature (Batlle and Massague, 2019), which have been shown to play a role in immune suppression and are associated with poorer treatment responses. The specific genes associated with each of these 4 signatures are displayed in Supplementary Table S5.

### Gene set enrichment analysis

Gene set enrichment analysis (GSEA) was used to identify significantly enriched molecular signaling pathways in high-risk and low-risk LUAD patients. Based on the R limma package (Ritchie et al., 2015), a genome-wide differential expression analysis was first conducted between the high-risk and low-risk subgroups, with the resulting t-values serving as input variables for pathway analysis using the R clusterProfiler package (Wu D. et al., 2021). Signaling pathways from the KEGG and GO BP databases were used for background annotation. For gene sets from specific immune cells and molecular signatures, we used the single sample GSEA (ssGSEA) method (Hanzelmann et al., 2013) to enrich each LUAD sample and obtain corresponding enrichment scores.

### Detection of tumor mutational signatures

Mutational signatures refer to the changes in a genome that occurs as it is continuously subjected to endogenous and exogenous DNA damage during cellular growth, ultimately resulting in distinct genomic markers (Koh et al., 2021). Using mutational profiles from the LUAD discovery dataset, we employed Bayesian nonnegative matrix factorization (NMF) method to extract potential mutational signatures and their activity, and analyzed their enrichment differences across distinct risk subpopulations. The NMF approach automates the identification of potential mutational signatures without requiring manual inspection. In particular, the mutational feature matrix ***A*** was decomposed into two nonnegative matrices ***W*** and ***H*** (i.e., ***A*** ≈ ***W*** × ***H***): the ***W*** matrix representing identified mutational signatures, and the ***H*** matrix representing corresponding mutational activity. Finally, all identified mutational signatures were aligned with already annotated signatures in the COSMIC database (Alexandrov et al., 2013) based on cosine similarity to determine the final information. All relevant analyses were completed with R maftools package (Mayakonda et al., 2018).

### Determination of significantly mutated genes

Significantly mutated genes (SMGs) refer to genes with mutation frequencies significantly higher than the background mutation frequency. These mutations are typically considered in combination with somatic single nucleotide variants (SNVs) and insertions/deletions (INDELs). The MutSigCV algorithm (Lawrence et al., 2013) was used for identifying SMGs based on LUAD mutational profiles. MutSigCV establishes a background mutation process model that functions during tumor formation. By analyzing the mutations of each gene, it determines which genes have a higher mutation frequency than expected given the background model. First, mutation data from multiple tumor samples are aggregated together; then, the scores and *P* values for each gene are calculated. A significance threshold is selected to control the false discovery rate, where genes exceeding this threshold are considered to be SMGs.

### Processing of LUAD intratumor microbiome data

The study by Poore et al. (2020) provides normalized intratumor microbial abundance data, which is derived from treatment-naive whole genome and transcriptome sequencing of TCGA LUAD samples for microbial reads and subsequent quantification of microbial abundances. The investigation of intratumoral microbial diversity encompasses two aspects: firstly, the alpha diversity that reflects the abundance and diversity of microbial communities, representing the ecological community structure. This aspect can be assessed using various indexes such as the Shannon index (diversity accounting for richness and evenness), Richness index (number of unique OTUs), ACE index (estimating species richness with rare taxa), and Chao1 index (species richness with unseen species). Secondly, beta diversity involves comparative analysis of microbial community composition across groups, evaluated using Bray-Curtis distances and visualized via PCoA plots using GUniFrac and ggplot2.

### Statistical analysis

The majority of the analyses in this study were completed using R software (version 4.0.2), while some specific analyses such as the identification of SMGs were implemented using Python. The Kaplan-Meier method was employed to plot survival curves between different LUAD subgroups, and the Log-rank test method was used to detect significant differences in survival. Multivariate regression models were used to adjust for confounding factors such as age, gender, stage, and smoking history; among these factors, some had missing values, and we removed samples with any missing data. *P* values less than 0.05 were considered statistically significant. Hypothesis tests were conducted to assess the association of continuous and categorical variables with two risk subgroups using the non-parametric tests (i.e., Wilcoxon rank-sum test and Kruskal-Wallis H test) and Fisher exact test, respectively. We utilized the Hosmer-Lemeshow (HL) test function from the R rms package and the PredictABEL package to calculate the calibration of all Cox regression models included in this study. A non-significant HL test result (*P* ≥ 0.05) indicates good model calibration.

## Results

### Construction of the FAT1 mutation-related risk signature

**FIGURE 2:**
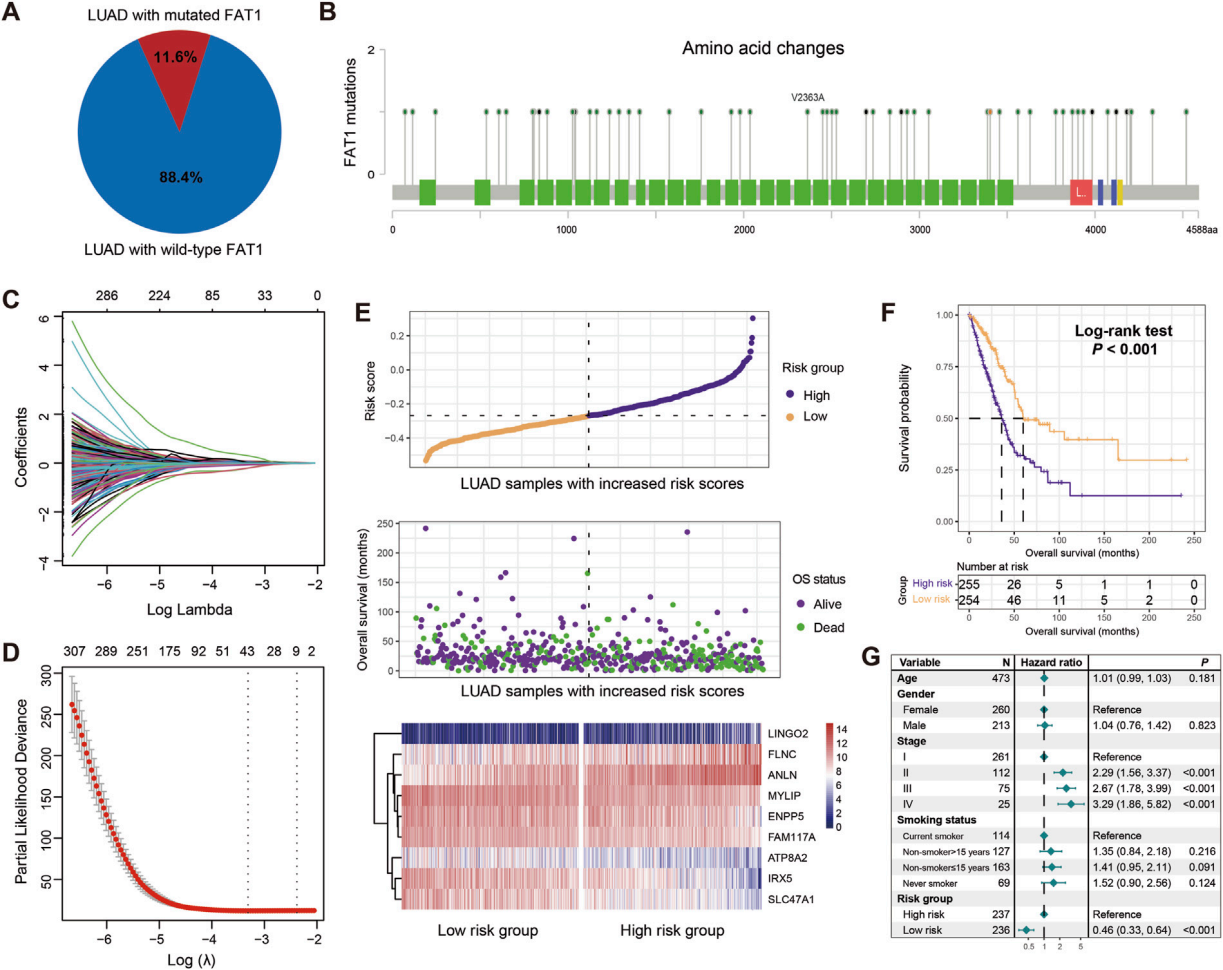
Construction of a risk signature associated with FAT1 mutations in LUAD based on the discovery dataset. **(A)** Proportion of FAT1 mutation and wild-type LUAD patients in the discovery dataset. **(B)** Changes in all amino acids resulting from FAT1 mutations. Green blocks represent amino acid changes induced by FAT1 missense mutations, blue blocks represent frame shift mutations, red blocks represent nonsense mutations, and yellow blocks represent splice site mutations. **(C)** Lasso regression profiles of associations of lambda and coefficients. **(D)** Variation in partial likelihood deviance (PLD) with respect to log lambda changes. The red dots indicate the detailed partial likelihood of deviance values, the gray lines indicate the standard error (SE), the two vertical dotted lines on the left and right indicate the optimal gene combination with 1-SE criteria and minimum criteria, separately. **(E)** Distribution of risk scores in LUAD samples and their association with survival. Differential expression of nine FAT1 mutation-related genes in high-risk and low-risk subgroups. **(F)** Kaplan-Meier survival curves stratified by high- and low-risk populations. **(G)** Multivariate Cox regression model incorporating age, gender, clinical stage, and smoking status to obtain the real association between risk signature and LUAD prognosis.

After excluding samples without transcriptome expression profiles or prognostic information, a total of 2,528 patients with LUAD from 12 datasets were included in this study. TCGA LUAD cohort was chosen due to its largest sample size and comprehensive multi-omics information, which were used as the discovery dataset for constructing a FAT1 mutation-related risk signature and subsequent multi-dimensional molecular feature analyses. In total, 11.6% of all LUAD patients carried FAT1 mutations (Figure 2A). The detailed amino acid changes induced by FAT1 mutations are shown in Figure 2B. A whole-genome differential analysis of FAT1 mutation and wild-type LUAD patients was performed to identify FAT1 mutation-related genes, resulting in a total of 1,530 genes exhibiting differential expression levels between the two groups (Supplementary Table S6). Subsequently, univariate Cox regression was utilized to explore the prognostic genes among the differentially expressed genes, with a total of 558 genes showing significance (all *P* < 0.05; Supplementary Table S7). Further, based on 10-fold cross-validation Lasso-Cox regression, we identified the genes that contributed most to prognoses. The Lasso coefficient plot for different gene combinations versus log(λ) is presented in Figure 2C. The smallest partial likelihood deviance was achieved when there are 9 specific genes (Figure 2D). Therefore, we selected these 9 FAT1 mutation-related genes for constructing a LUAD molecular risk signature.

The identified 9 FAT1 mutation-related genes, including IRX5, SLC47A1, MYLIP, ATP8A2, ENPP5, FAM117A, LINGO2, FLNC, and ANLN, are shown in Supplementary Table S8 with their contribution to the prognosis of LUAD. For each patient, we calculated a risk score based on each gene and the corresponding prognostic coefficients determined by the Lasso regression (Figure 2E). Furthermore, the associations of high-risk and low-risk patients with survival time and status are presented in Figure 2E. Additionally, the expression levels of these 9 genes across the high-risk and low-risk subgroups are also shown (Figure 2E).

To evaluate the prognostic capacity of our risk signature, we divided all patients into high-risk (N = 255) and low-risk (N = 254) groups based on the median risk score. The Kaplan-Meier survival analysis reveal that compared to high-risk patients, low-risk patients showed significantly increased overall survival (Log-rank test, *P* < 0.001; Figure 2F), and this association remained significant after adjusting for age, gender, stage, and smoking history in a multivariable Cox regression model (HR: 0.46, 95% CI: 0.33–0.64, *P* < 0.001; HL test, χ2 = 2.109, *P* = 0.147; Figure 2G). The above results indicate that the LUAD molecular signature constructed based on 9 FAT1 mutation-related genes harbor prognostic significance.

### Verification of the risk signature

**FIGURE 3:**
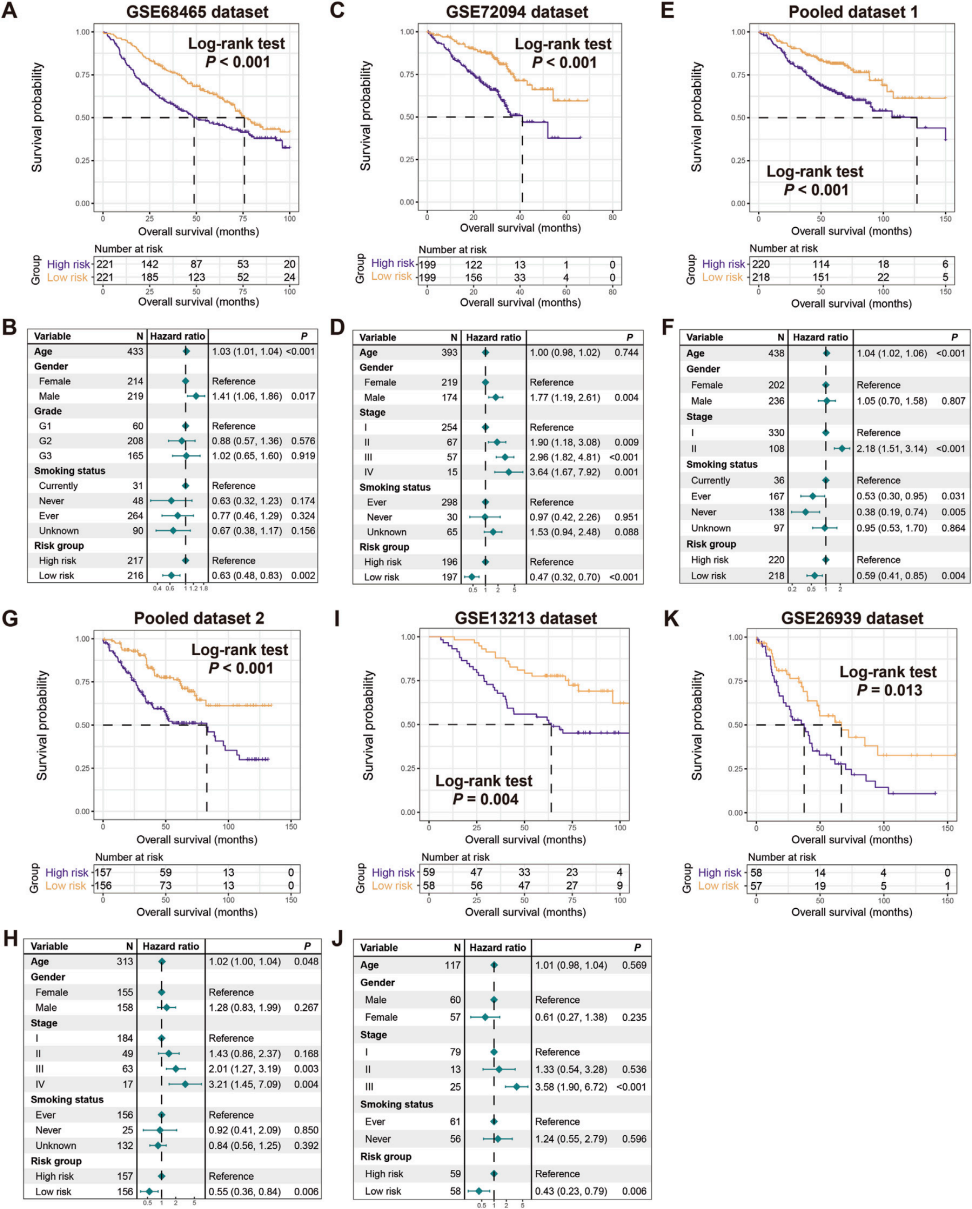
Validation of the constructed risk signature. Kaplan-Meier survival curves divided with low- and high-risk LUAD patients in **(A)** GSE68465, **(C)** GSE72094, **(E)** pooled dataset 1, **(G)** pooled dataset 2, **(I)** GSE13213, and **(K)** GSE26939. Multivariate Cox regression models of the associations between FAT1 mutation risk signature and LUAD prognosis were performed in **(B)** GSE68465, **(D)** GSE72094, **(F)** pooled dataset 1, **(H)** pooled dataset 2, and **(J)** GSE13213.

To validate the prognostic capacity of the determined risk signature, we utilized 8 additional LUAD datasets. In 6 datasets of GSE68465, GSE72094, pooled dataset 1, pooled dataset 2, GSE13213, and GSE26939, we also observed that patients in the low-risk subgroups had significantly better prognoses compared to those in the high-risk subgroups (Log-rank test, *P* < 0.001 for GSE68465, GSE72094, pooled dataset 1, and pooled dataset 2, *P* = 0.004 for GSE13213, and *P* = 0.013 for GSE26939; Figures 3A,C,E,G,I,K). Multivariable Cox regression models incorporating clinical confounders (e.g., age, gender, clinical stage, and smoking history) further confirm the independent prognostic power of this risk signature (GSE68465, HR, 0.63, 95% CI, 0.48–0.83, *P* = 0.002, HL test χ2 = 0.779, *P* = 0.377; GSE72094, HR, 0.47, 95% CI, 0.32–0.70, *P* < 0.001, HL test χ2 = 0.156, *P* = 0.693; pooled dataset 1, HR, 0.59, 95% CI, 0.41–0.85, *P* = 0.004, HL test χ2 = 0.228, *P* = 0.572; pooled dataset 2, HR, 0.55, 95% CI, 0.36–0.84, *P* = 0.006, HL test χ2 = 0.656, *P* = 0.433; GSE13213, HR, 0.43, 95% CI, 0.23–0.79, *P* = 0.006, HL test χ2 = 2.008, *P* = 0.159; Figures 3B,D,F,H,J). However, due to the lack of available clinical pathology information in GSE26939 dataset, the multifactorial correction analysis was not performed. Further analysis reveals that elevated risk scores were significantly enriched in LUAD patients with advanced stages (TCGA, GSE13213, GSE72094, pooled cohort 1 and 2, Kruskal-Wallis H test all *P* < 0.05; Supplementary Figures S1A–E) or worse grades (GSE68465, Kruskal-Wallis H test *P* < 0.001; Supplementary Figure S1F). The above results further validate the prognostic power of the LUAD FAT1 mutation signature.

In the remaining two datasets of GSE81089 (N = 106) and GSE11969 (N = 90), survival analysis reveals that patients in the low-risk subgroups harbored favorable prognostic tendencies compared to the high-risk subgroups. However, no statistically significant differences are achieved due to the relatively small sample size (Log-rank test, both *P* > 0.05; Supplementary Figure S2), which may have been the primary cause.

### FAT1 mutation risk signature associated with immune infiltration and immunogenicity

**FIGURE 4:**
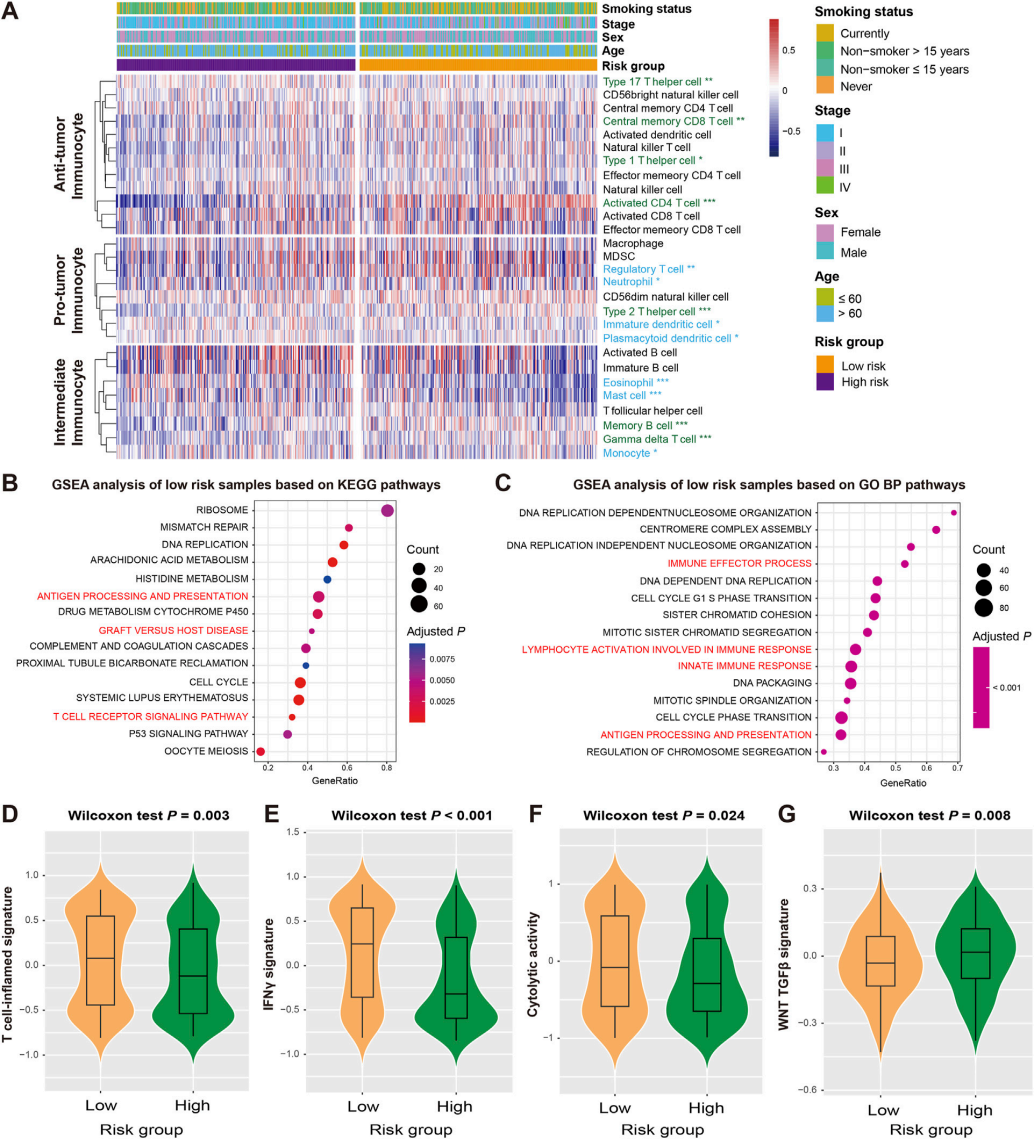
Association of the FAT1 mutation risk signature with immunocyte infiltration and tumor immunogenicity. **(A)** Infiltration proportion of distinct immunocytes in low-versus high-risk LUAD subgroups. Immunocytes highlighted with green represent its infiltration was enhanced in low-risk patients, whereas the blue represent the infiltration was decreased in low-risk patients. GSEA analyses of low-risk patients were performed based on **(B)** KEGG and **(C)** GO BP databases. Pathways highlighted with red are immune response-related. Distinct enrichment scores of **(D)** T cell-inflamed signature, **(E)** IFNγ signature, **(F)** cytolytic activity signature, and **(G)** WNT TGF-β signature in low- and high-risk LUAD subgroups.

Recent studies have reported the roles of FAT1 and its mutations in immunoregulation and cancer treatment. In light of this, we conducted an analysis of the relationship between the FAT1 mutation-related signature and immunological molecular signatures. Using the ssGSEA method and LUAD transcriptome profiling, we estimated the abundance of 28 immune cell subtypes infiltrating the tumor microenvironment in the discovery dataset and analyzed their differences between high-risk and low-risk patients with LUAD (Figure 4A). Our results demonstrate that anti-tumor immune response cells (e.g., activated CD4 T cells, central memory CD8 T cells, and type 1/17 helper cells) exhibited higher levels of infiltration in low-risk patients, consistent with lower levels of immune suppressive cells (e.g., immature dendritic cells, neutrophils, plasmacytoid dendritic cells, and regulatory T cells) in these patients (Wilcoxon rank-sum test, all *P* < 0.05). Additionally, intermediate immunocytes displayed different infiltration patterns in the low-risk group, including increased infiltration of gamma delta T cells and memory B cells, and decreased infiltration of eosinophils and monocytes (all *P* < 0.05). We also employed two methods (i.e., CIBERSORT and TIMER) to assess immune cell infiltration in LUAD, and similar to what was observed, higher levels of immune response cells represented by CD8 T cells were noticed in the low-risk group (Supplementary Figures S3A, B).

The analysis of gene expression profiles using the GSEA approach was used to identify potential signaling pathways that may be related to the risk signature, and further elucidate its roles in regulating prognosis. By comparing the well-known databases, we found that pathways related to immune regulation and activation in KEGG (e.g., antigen processing and presentation, graft versus host disease, and T cell receptor signaling pathway) and GO BP (e.g., immune effector process, innate immune response, and lymphocyte activation involved in immune response), were significantly enriched in the low-risk group (all adjusted *P* < 0.01; Figures 4B,C). To validate the pathway results, we also performed GSEA analysis based on HALLMARK (Supplementary Figure S4A) and REACTOME databases (Supplementary Figure S4B), the immune response relevant pathways were also observed in low-risk LUAD patients.

Several molecular signatures proposed recently have been shown to be related to the immunogenicity, immune regulation, and therapeutic effects of tumors. Therefore, we also analyzed their differential enrichment in subgroups of high and low risk. Our results indicate that the low-risk group harbored significantly enhanced enrichment scores for T cell-inflamed signature, IFNγ signature, and cytolytic activity signature (Wilcoxon rank-sum test, all *P* < 0.05; Figures 4D–F), whereas the WNT TGF-β signature with immunosuppressive properties was absent in this group (Wilcoxon rank-sum test, *P* = 0.008; Figure 4G).

The distinct distribution of immune checkpoint genes between two risk subgroups was evaluated. Several immune checkpoints (e.g., CD27, CD40LG, CEACAM1, and LAG3) showed markedly elevated expression in the low-risk group, while several (e.g., CD274, ICOS, TNFRSF4, and TNFRSF9) showed reduced levels (Wilcoxon rank-sum test, all *P* < 0.05; Supplementary Figure S5). The above results indicate that the LUAD low-risk subgroup may have better immune infiltration.

### Genomic mutational traits linked with the FAT1 mutation risk signature

**FIGURE 5:**
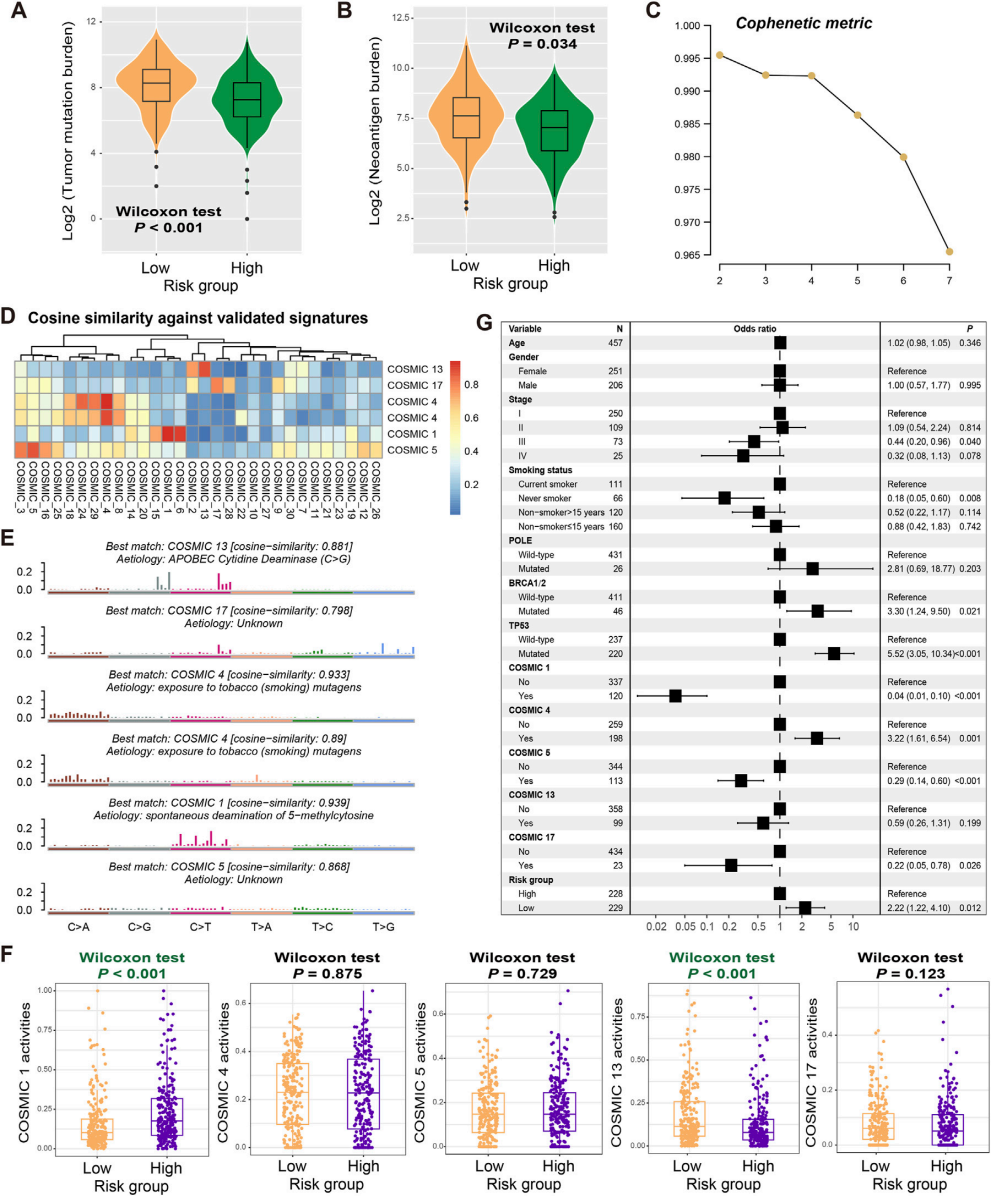
Mutational features associated with the constructed risk signature. Associations of the determined FAT1 mutation risk signature with **(A)** TMB and **(B)** NB. **(C)** Associations of the cophenetic metric with extracted LUAD mutational signature numbers. **(D)** The detected six mutational signatures versus well-known COSMIC signatures using the cosine similarity. **(E)** Detailed mutational features of the detected six mutational signatures. **(F)** Distinct mutational activities of five signatures between low- and high-risk patients. **(G)** Multivariate Logistic regression was conducted with age, sex, stage, smoking status, detected mutational signatures, and DNA repair gene mutations taken into account to acquire the connection between FAT1 mutation risk signature and TMB.

The genetic mutational burden plays a pivotal role in the evaluation of tumor prognosis and predicting immunotherapy efficacy. Therefore, we explored the relationship between identified risk signature and tumor mutational burden (TMB) and neoantigen burden (NB). Based on the somatic mutation data from the discovery dataset, we calculated TMB for each LUAD patient and found that TMB was significantly higher in low-risk patients than in high-risk patients (Wilcoxon rank-sum test, *P* < 0.001; Figure 5A). Similar results were also observed in NB (Wilcoxon rank-sum test, *P* = 0.034; Figure 5B), indicating that the mutation load in low-risk populations is higher.

Mutational signatures, which are the specific markers of genomic damage caused by endogenous or exogenous insults, play a crucial role in cancer prognosis and treatment efficacy evaluation. We utilized the NMF method to decompose the LUAD mutation feature matrix and extracted possible mutation signatures operative in the genome. From the cophenetic metric map, it was observed that the line dropped fastest when the number of signatures was 6, suggesting that there may be 6 mutational signatures in the LUAD mutational data (Figure 5C). By comparing these possible signatures with those annotated in the COSMIC database using cosine similarity (Figure 5D), we noticed that two signatures were repeating and ultimately identified COSMIC 1 (associated with age at diagnosis), COSMIC 4 (associated with tobacco smoking), COSMIC 5 (unknown aetiology), COSMIC 13 (attributed to the activity of the AID/APOBEC family), and COSMIC 17 (unknown aetiology). Their detailed mutation patterns are presented in Figure 5E. The specific mutation activities of these signatures across all LUAD patients were calculated and displayed in Supplementary Figure S6 and Supplementary Table S9. Further analysis reveals that low-risk patients exhibited significantly lower COSMIC 1 mutation activity and higher COSMIC 13 mutation activity (Wilcoxon rank-sum test, both *P* < 0.001; Figure 5F).

To investigate whether the high TMB observed in low-risk patients was influenced by other confounding factors, we included common clinical variables (i.e., age, sex, stage, and smoking history), identified mutational signatures (i.e., COSMIC 1, 4, 5, 13, and 17), and classical DNA damage repair gene (e.g., POLE, BRCA1/2, and TP53) mutations in a multivariate logistic regression model. The results show that there remained a statistically significant correlation between low-risk scores and elevated TMB levels (OR: 2.22, 95% CI: 1.22–4.10, *P* = 0.012; Figure 5G).

**FIGURE 6:**
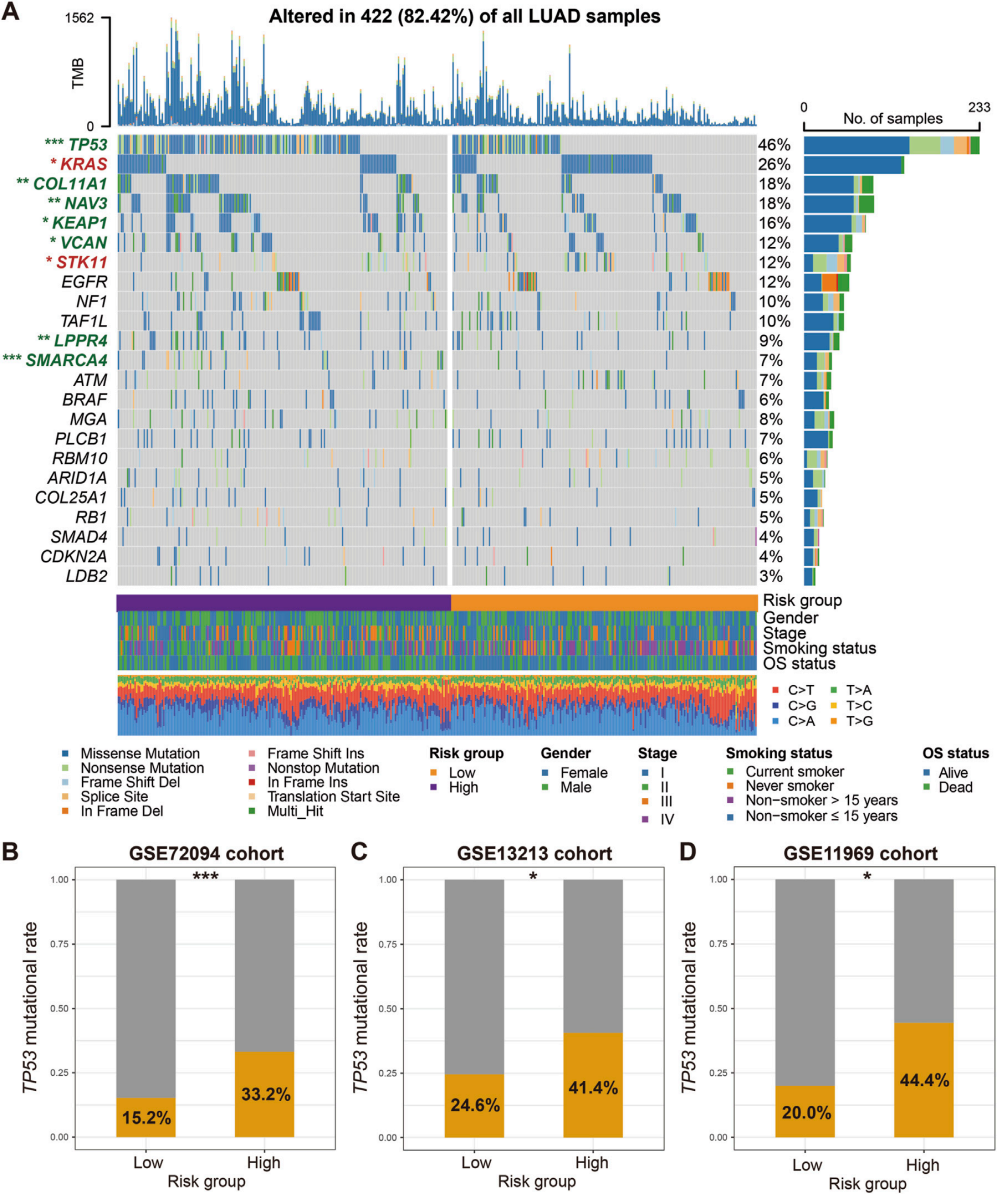
Identification of LUAD SMGs and their distinct mutation frequencies in two risk subgroups. **(A)** Waterfall plot representation of 23 SMGs determined from LUAD somatic mutational data in low-versus high-risk patients. SMGs highlighted with green exhibit the significantly decreased mutation frequencies in the low-risk group, whereas SMGs highlighted with red exhibit the increased mutation frequencies in the low-risk group. Validation of the association of TP53 mutation frequencies with two risk groups in **(B)** GSE72094, **(C)** GSE13213, and **(D)** GSE11969 datasets. *P < 0.05, **P < 0.01, ***P < 0.001.

Based on the mutational landscape and the MutSigCV algorithm applied to the discovery dataset, a total of 23 significantly mutated genes (SMGs) were identified. The waterfall plot of SMGs between low-risk and high-risk groups (Figure 6A) show significant differences in mutation frequency at TP53 [68 of 228 (29.8%) vs. 152 of 229 (66.4%); *P* < 0.001], KRAS [72 of 228 (31.6%) vs. 53 of 229 (23.1%); *P* = 0.047], COL11A1 [32 of 228 (14.0%) vs. 59 of 229 (25.8%); *P* = 0.002], NAV3 [37 of 228 (16.2%) vs. 62 of 229 (27.1%); *P* = 0.006], KEAP1 [33 of 228 (14.5%) vs. 50 of 229 (21.8%); *P* = 0.048], VCAN [26 of 228 (11.4%) vs. 43 of 229 (18.8%); *P* = 0.036], STK11 [45 of 228 (19.7%) vs. 28 of 229 (12.2%); *P* = 0.031], LPPR4 [15 of 228 (6.6%) vs. 35 of 229 (15.3%); *P* = 0.004], and SMARCA4 [5 of 228 (2.2%) vs. 39 of 229 (17.0%); *P* < 0.001]. Among the aforementioned SMGs, seven genes (TP53, COL11A1, NAV3, KEAP1, VCAN, LPPR4, and SMARCA4) exhibited decreased mutation frequencies in the low-risk group, while KRAS and STK11 two genes showed increased mutation frequencies. In addition, the correlation between low-risk scores and lower TP53 mutation frequencies was validated in three independent LUAD datasets (GSE72094: 15.2% vs. 33.2%, *P* < 0.001; GSE13213: 24.6% vs. 41.4%, *P* = 0.077; GSE11969: 20.0% vs. 44.4%, *P* = 0.023; Figures 6B–D). Other SMG mutation information was not available in the validation dataset.

The mutation frequencies displayed in Figure 6A represent the proportion of samples carrying a mutation in each SMG across the entire TCGA LUAD cohort with available mutation data. However, the subsequent analysis comparing mutation frequencies between the high-risk and low-risk subgroups utilized a subset of these samples. This subset included only samples with both transcriptomic expression data (required to calculate the FAT1 mutation risk signature score and assign risk groups) and somatic mutation data. Consequently, the total sample size for the subgroup comparison (N = 457) is smaller than that used to establish the overall mutation frequencies in Figure 6A (N = 509). This difference in underlying sample cohorts (full mutation cohort vs. mutation + expression intersection cohort) accounts for the observed numerical discrepancies in mutation rates.

### Association of LUAD risk signature with intratumor microbial diversities

**FIGURE 7:**
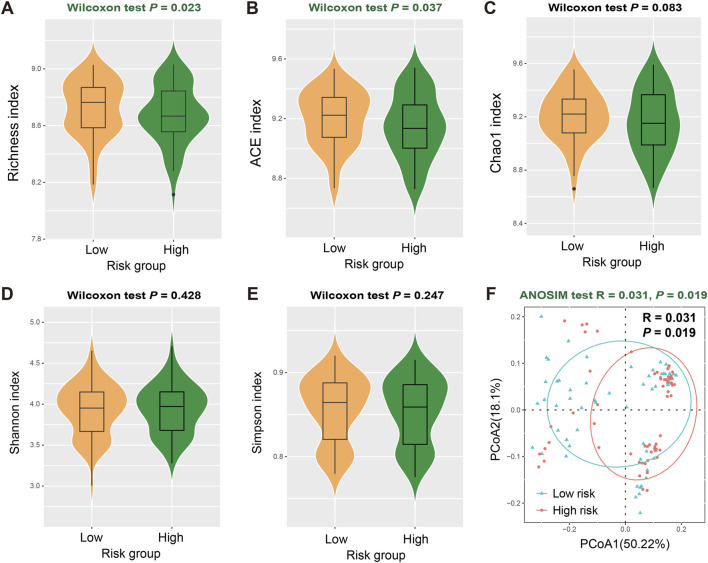
Association of FAT1 mutation risk signature with intratumor microbial diversities. Distinct intratumor microbial α diversity indexes including **(A)** Richness, **(B)** ACE, **(C)** Chao1, **(D)** Shannon, and **(E)** Simpson index in low- and high-risk LUAD patients. **(F)** Distinct β diversity between two risk subgroups evaluated with the ANOSIM test.

There is a growing body of evidence that suggests the role of intratumor microbes in regulating the tumor microenvironment homeostasis and predicting disease survival risks. Therefore, we analyzed the correlation between identified LUAD risk signatures and intratumor microbial diversities. In terms of α diversity, the low-risk group exhibited significantly higher Richness (Wilcoxon rank-sum test, *P* = 0.023; Figure 7A) and ACE indices (Wilcoxon rank-sum test, *P* = 0.037; Figure 7B) compared with high-risk group, while Chao1, Shannon, and Simpson indexes were not detected significant differences between the two risk subpopulations (Wilcoxon rank-sum test, all *P* > 0.05; Figures 7C–E). Similarly, we also observed a significant increase in β diversities in the low-risk populations (ANOSIM test, R = 0.031, *P* = 0.019; Figure 7F). The evidence above suggests that individuals with low-risk LUAD exhibited elevated levels of diversity in the intratumoral microbiome.

### The role of the FAT1 mutation risk signature in predicting immune response in cancer treatment

**FIGURE 8:**
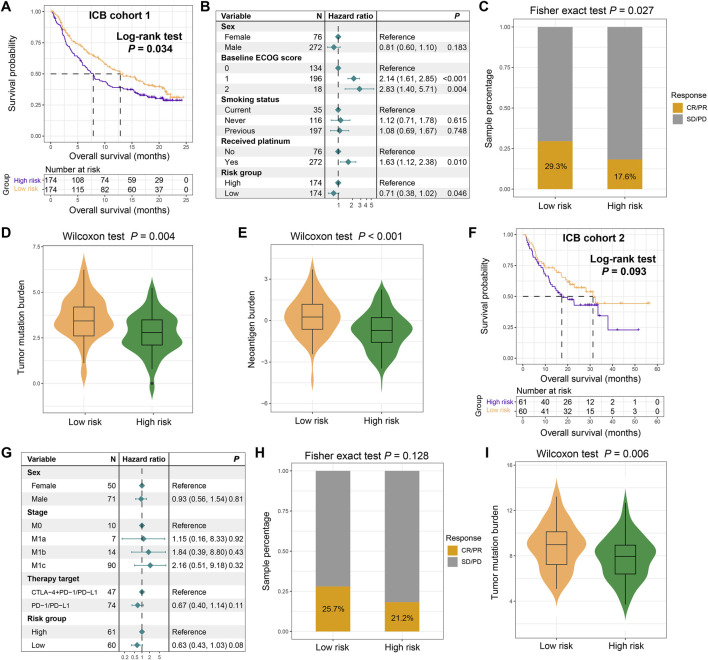
The constructed FAT1 mutation risk signature for assessing immunotherapeutic response. **(A)** Immunotherapeutic survival curves for low- and high-risk patients in the ICB cohort 1. **(B)** Multivariable Cox regression analysis showing the association between the FAT1 mutation risk signature and immunotherapeutic survival, adjusted for clinical covariates. **(C)** Distinct immunotherapeutic response rates in two risk subpopulations. **(D)** TMB and **(E)** NB levels in low- and high-risk subgroups in the ICB cohort 1. **(F)** Immunotherapeutic survival curves for low- and high-risk patients in the ICB cohort 2. **(G)** Multivariable Cox regression analysis showing the association between the FAT1 mutation risk signature and immunotherapeutic survival, adjusted for clinical covariates in the ICB cohort 2. **(H)** Distinct immunotherapeutic response rates in two risk subpopulations in the ICB cohort 2. **(I)** TMB levels in low- and high-risk subgroups in the ICB cohort 2.

Our previous findings indicated that the FAT1 mutation risk signature harbored strong correlations with tumor immunogenicity and related features, thus we hypothesize that this risk signature might play a role in evaluating the efficacy of immune therapy. Based on two ICB treatment cohorts collected, we validated this hypothesis. In the ICB cohort 1, Kaplan-Meier survival analysis reveal that low-risk patients exhibited significantly prolonged survival following immunotherapy (Log-rank test, *P* = 0.034; Figure 8A). This association remained statistically significant after adjusting for gender, ECOG score, smoking status, and platinum treatment status in a multivariable Cox regression model (HR: 0.71, 95% CI: 0.38–1.02, *P* = 0.046; Figure 8B). Consistently, we also observed increased ICB treatment response (complete response and partial response) rates among the low-risk subpopulation (29.3% vs. 17.6%, Fisher exact test, *P* = 0.027; Figure 8C). Subsequent mutation load analysis demonstrates that low-risk scores were significantly associated with elevated TMB (Wilcoxon rank-sum test, *P* = 0.004; Figure 8D) and NB (Wilcoxon rank-sum test, *P* < 0.001; Figure 8E), consistent with our previous findings. In the ICB cohort 2, we observed that low-risk individuals still exhibited improved immune therapy prognosis, although this result did not reach statistical significance (Log-rank test, *P* = 0.093; Figure 8F). A multivariate Cox regression model including gender, clinical stage, and treatment type further confirmed this trend (HR: 0.63, 95% CI: 0.43–1.03, *P* = 0.08; Figure 8G). Similarly, a higher treatment response was observed in the low-risk subpopulation compared to the high-risk group (25.7% vs. 21.2%, Fisher exact test, *P* = 0.128; Figure 8H). Among this cohort, high TMB was also shown to be correlated with low-risk scores (Wilcoxon rank-sum test, *P* = 0.006; Figure 8I). The above results indicate that the LUAD low-risk subgroup may achieve a higher response rate and better survival when treated with ICB therapy.

## Discussion

By integrating multi-omics data and clinical information of LUAD patients, this study constructed a FAT1 mutation-related signature for evaluating survival risk, immunogenicity and immunotherapy effect. The reliability and stability of the signature were confirmed by validating it across multiple independent datasets. Prospective studies are needed, but these findings offer a basis for future clinical trials and therapeutic strategies in LUAD.

Recent studies have reported on the roles of FAT1 in immune regulation and immunotherapy. A notable study by Feng et al. indicated a high mutation frequency of FAT1 in NSCLC (Feng et al., 2022), and they simultaneously discovered that patients harboring FAT1 mutations exhibited significantly increased infiltration of activated dendritic cells and extended progression-free survival from ICB treatment. Another study on NSCLC further validated these findings and pointed out that FAT1 mutations correlated with higher TMB and better response to ICB treatment (Fang et al., 2019). Zhang et al., by integrating somatic mutation data and clinical information from 631 melanoma samples, discovered that FAT1 mutations in this tumor type could also predict better immunotherapy responses, simultaneously associating with an activated immune microenvironment and immune signaling pathways (Zhang et al., 2022). Furthermore, FAT1 has been shown to regulate various canonical signaling pathways, including Hippo, WNT/β-catenin, TGF-β, and PI3K/AKT, all of which have been proven to be relevant to immune regulation (Chen Z. G. et al., 2022), indirectly substantiating FAT1’s regulatory role in the tumor immune system. Several non-oncological studies have also highlighted the significance of FAT1 in the immune system. Studies on FAT1 transgenic mice revealed downregulation of TNF-α, IL-6, NF-kB, and CCL2 gene expression compared to non-transgenic wild-type mice, suggesting that FAT1 overexpression may downregulate these inflammatory cytokines/chemokines (Fang et al., 2018). Conversely, mutated FAT1 may upregulate growth factors and pro-inflammatory factors such as TGFB1, IL-6, and FGF2 (Chen Y. H. et al., 2022). This mechanism might be mediated by YAP1, which is activated by FAT1 inactivation (Martin et al., 2018). Activated YAP1 has been found to upregulate CCL2 in endothelial cells (Xu et al., 2016). The above findings underscore the vital roles played by FAT1 and its mutations in immune regulation and tumor immunotherapy.

Considering the important roles of FAT1 in tumor progression and treatment, this study developed a mutation-associated risk signature for LUAD patients. We further explored the impact of FAT1 mutations on signature risk scores and found that patients with this gene mutations had markedly higher risk scores (Wilcoxon rank-sum test, *P* < 0.001; Supplementary Figure S7A), suggesting that FAT1 mutations may be associated with poor prognosis of LUAD, although its prognostic effect was not observed in the discovery dataset (Log-rank test, *P* = 0.763; Supplementary Figure S7B). In subsequent adjusted analysis, in addition to clinical factors, we also included the FAT1 mutation status in the multivariate Cox regression model, and the results showed that patients with low-risk scores still exhibited significantly prolonged survival (HR: 0.46, 95% CI: 0.33–0.65, *P* < 0.001; Supplementary Figure S7C), indicating that this risk signature can be employed as an independent prognostic predictor.

The FAT1 mutation risk signature constructed in this study encompassed nine relevant genes. LINGO2, a stem cell-related marker, has been demonstrated to correlate with worse prognosis in gastric cancer (GC) patients (Jo et al., 2019). Recently, a study reported that FLNC effectively suppressed GC progression by promoting the overexpression of TRIM54 (Cao et al., 2022). In this study, high expression of FLNC was associated with poor prognosis in LUAD. Through literature review, we found that in prostate cancer (Amaro et al., 2014) and non-small cell lung cancer (Ding et al., 2022), patients with high FLNC expression also showed poor prognosis. These evidences indicate that due to tumor heterogeneity, FLNC exhibits different roles through distinct regulatory mechanisms in various tumor types. The oncogene ANLN could be targeted and enhanced by USP10, leading to inferior prognosis for patients with esophageal squamous cell carcinoma (Cao et al., 2023). MYLIP, identified as a tumor suppressor gene, its high expression effectively inhibited the proliferative, migratory, and invasive capabilities of lung cancer (Wang et al., 2021). FAM117A served as a known prognostic marker for LUAD and is capable of predicting the therapeutic efficacy of cell cycle inhibitors (Wu et al., 2022). A risk model incorporating ATP8A2 has been developed to predict the survival of patients with luminal A breast cancer (Chen Z. G. et al., 2022). IRX5 promoted the metastasis of colorectal cancer by regulating the RHOA pathway (Zhu et al., 2019). Notably, this study revealed that in LUAD, patients with high IRX5 expression showed a better prognosis. Additionally, the study by Yu et al. further validated the association between IRX5 and favorable prognosis in LUAD (Yu et al., 2021), suggesting that IRX5 may also exert different biological functions depending on tumor types. Collectively, these findings further reinforce the reliability of our risk signature in assessing the prognosis of LUAD.

Existing evidence also supported the roles of FAT1-related genes in cancer biology and immunogenicity. ENPP5 regulates nucleotide metabolism and has been implicated in purinergic signaling, which modulates immune cell activity (e.g., T-cell exhaustion via adenosine production). Recent studies link ENPP family members to immunosuppressive microenvironments in NSCLC (Borza et al., 2022). LINGO2 is a stem cell marker associated with poor prognosis in gastric cancer (Jo et al., 2019). While its direct role in LUAD is underexplored, LINGO2 overexpression correlates with EMT and immune evasion in pan-cancer analyses. IRX5 promotes metastasis via RHOA signaling in colorectal cancer (Zhu et al., 2019) and regulates WNT/β-catenin pathways, which intersect with FAT1-mediated Hippo signaling (Holmquist Mengelbier et al., 2019). FLNC suppresses gastric cancer progression by stabilizing TRIM54 (Cao et al., 2022) and is linked to cytoskeletal remodeling, a process modulated by FAT1 in EMT (Guyon et al., 2003).

Through multi-dimensional immunological analysis, this study highlighted that the low-risk subgroup of LUAD displayed a more favorable immune microenvironment and enhanced immunological features. The enhanced immunogenicity observed in low-risk patients was attributed to the interplay among multiple immune factors. For instance, we found that low-risk patients had a higher TMB, lower enrichment of COSMIC 1 signature, and higher enrichment of COSMIC 13 signature. Previous studies have shown that patients harboring COSMIC 1 and COSMIC 13 signatures exhibited low TMB (Chong et al., 2021) and high TMB (Gupta et al., 2023), respectively, aligning with the findings of this study. Furthermore, patients with a high mutation burden were more prone to activated immune cell infiltration and immune signaling pathways (Hu et al., 2021), leading to a more favorable immune microenvironment. It should acknowledge that TMB is not a standalone predictor. Factors such as antigen presentation deficits, HLA loss, and immune evasion mechanisms can limit the effectiveness of immunotherapy despite elevated TMB. The multiple immune factors carried by low-risk LUAD patients synergistically contribute to their better immunogenicity and therapeutic outcomes.

Extensive knowledge has demonstrated the role of SMGs in prognosis, immune regulation, and evaluation of treatment effectiveness. LUAD patients with TP53 (Jiao et al., 2018), KEAP1 (Scalera et al., 2021), and SMARCA4 mutations (Schoenfeld et al., 2020) have been observed to have poor clinical prognoses, while in our study, these gene mutations significantly decreased in low-risk populations, which is consistent with the elevated survival rates observed in this subgroup. In addition, Dong et al. study demonstrated that patients with KRAS-mutated LUAD respond better to ICB treatment (Dong et al., 2017), and the low-risk subgroup exhibited a higher KRAS mutation frequency, which may be one of the reasons for the improved immunotherapy response. On the other hand, STK11 mutations, which have been reported to associate with poorer immunotherapeutic responses (Skoulidis et al., 2018), were also found to have a higher mutation frequency in the low-risk subgroup. We speculate that the multiple immune-stimulating factors in the low-risk group may have outweighed the inhibitory effects of STK11 mutations, leading to better immunotherapy responses in low-risk LUAD patients.

In our analysis of 32 immune checkpoints (including both co-inhibitory and co-stimulatory molecules), we observed that low-risk LUAD patients exhibited upregulated expression of immune activation markers (e.g., CD27, CD40LG, and LAG3) alongside downregulated immunosuppressive checkpoints (e.g., CD274/PD-L1 and TNFRSF9). These patterns reflect a complex immune regulatory landscape where enhanced T-cell priming [via CD40LG/CD40 interaction in antigen-presenting cells (Lu et al., 2000)] and memory T-cell activation [mediated by CD27/CD70 signaling (Borst et al., 2005)] may coexist with partial T-cell exhaustion mechanisms [suggested by elevated LAG3 (Andrews et al., 2024)]. Importantly, while PD-L1 downregulation in low-risk patients appears contradictory to conventional ICB response predictors, this aligns with recent findings that PD-L1-independent mechanisms [e.g., enhanced immunogenicity via APOBEC mutational signatures (Ma et al., 2023)] can drive ICB sensitivity. The observed patterns suggest a balanced yet active immune microenvironment in low-risk patients, where compensatory co-inhibitory signals may prevent overactivation while maintaining anti-tumor responses.

In addition to using the median to stratify the LUAD signature risk scores, we also utilized the surv_cutpoint function within the R survminer package to stratify LUAD patients and analyzed the survival differences between high- and low-risk subgroups across all datasets. The results indicated that there were still statistically significant differences in survival between high-risk and low-risk LUAD patients stratified using this method, with better prognosis observed in low-risk patients and poorer prognosis in high-risk patients (Supplementary Figure S8). Wang et al. study proposed the TMBcat method (Wang Y. et al., 2022), which is a minimal joint p-value criterion aimed at differentiating comprehensive therapeutic advantages and optimizing TMB categorization across distinct cancer cohorts, surpassing known benchmarks. However, TMB represents the total count of somatic mutations within a tumor patient’s tissue, which is a discrete numerical variable. In contrast, the signature risk score in our study is derived from gene expression and corresponding regression weights, making it a continuous numerical variable. Therefore, we believe that this cut-off determination method is not suitable for the context of our study.

In this study, we employed Lasso-Cox regression for feature gene selection and constructed a prognostic signature. We also applied five additional prognostic modeling approaches for gene selection and signature construction, including random survival forest (RSF) regression, elastic network (Enet) regression, ridge regression, partial least squares regression for Cox (plsRcox), and supervised principal components (SuperPC) regression. And we compared the performance among various methods using the C-index. We found that the use of different prognostic models had little impact on the final results (Supplementary Figure S9).

The purpose of the multivariate Cox regression model is to adjust for confounding factors in order to obtain more accurate results. In this study, we included age, gender, clinical stage, and smoking history in the multivariate Cox model for adjustment, aiming to reveal the true association between the FAT1 mutation signature and the prognosis of LUAD. For age and clinical stage, there is a clinical consensus that older age and advanced stage are associated with poorer prognosis in patients, making these two factors significant predictors of tumor prognosis. Therefore, we included them in the Cox regression model for adjustment. Regarding gender, several recently published studies have demonstrated significant differences in prognosis and prognostic indicators between male and female lung cancer patients (Guerreiro et al., 2023; Huh et al., 2024; Sachs et al., 2021), which is why we also included gender in the regression model. Smoking history plays a crucial role in the occurrence, progression, and prognosis of lung cancer, thus necessitating its adjustment in our analysis. Although these factors did not exhibit statistical significance in some of the Cox regression models in this study due to sample size limitations, we believe that adjusting for them is still necessary to obtain more accurate results. Recent published studies also aligned with our approach (Chen et al., 2019; Shen et al., 2020; Wu T. et al., 2021).

There are several limitations in this study. Firstly, the lack of LUAD immunotherapy cohorts with transcriptome gene expression data, somatic mutation data, and protein expression data prevents us from confirming the stability of FAT1 mutation-related signature in evaluating immunotherapy responses and comparing the differences with TMB and PD-L1 protein expression. Secondly, all the included LUAD datasets do not originate from the same platform, which may introduce a small amount of bias during data analysis. Thirdly, the potential biological mechanisms behind the various associations have not been explored and validated using functional experiments. It should also be noted that dataset-specific thresholds generated by the application of surv_cutpoint might impose limitations on the generalizability of the risk score across different cohorts.

## Conclusion

In summary, based on a large cohort of patients with LUAD, we have developed a molecular risk signature using transcriptome profiles associated with FAT1 mutations to assess patient prognosis and treatment response. Additionally, this study provides a potential molecular biomarker for clinical practice in LUAD.

## Data Availability

The datasets presented in this study can be found in online repositories. The names of the repository/repositories and accession number(s) can be found in the article/Supplementary Material.
