# Supplementary material for: FAT1 mutation-related signature predicts survival risk and tumor immunogenicity in lung adenocarcinoma
Source: Front Genet. 2025 Jul 2;16:1466484. doi: 10.3389/fgene.2025.1466484 (PMC12263358; doi:10.3389/fgene.2025.1466484)
Supplement: Supplementary file 1 [file DataSheet1.pdf]

Supplementary Figures

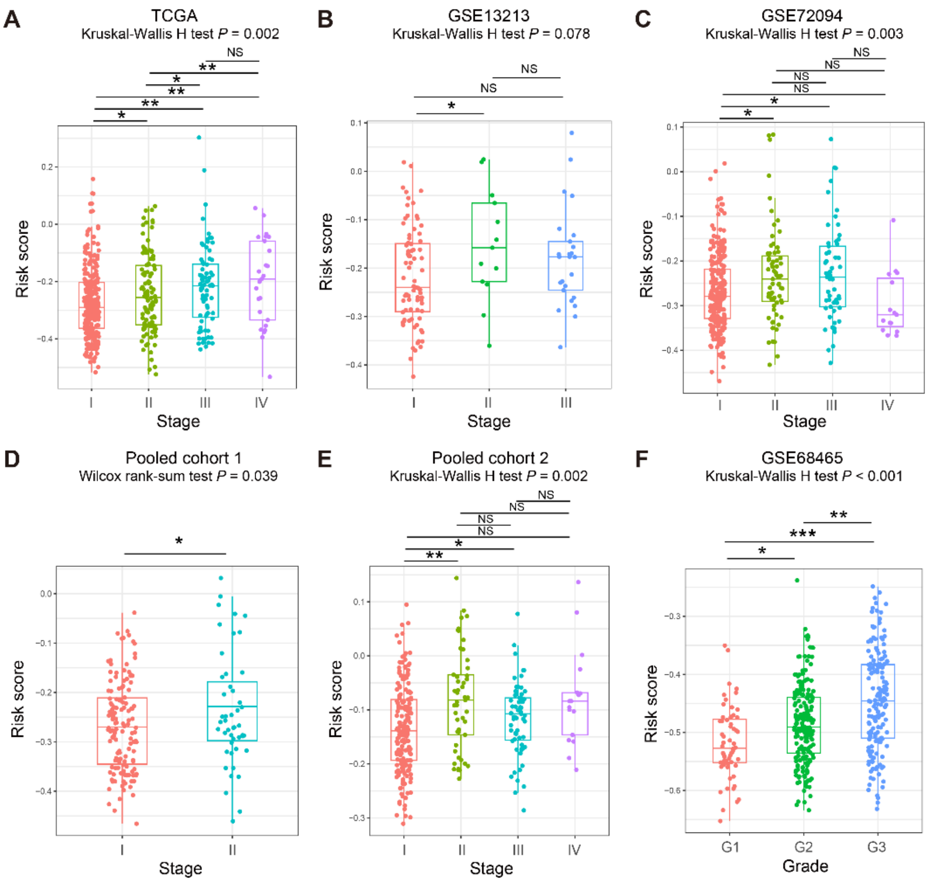

Figure S1. Distribution of the identified FAT1 mutation risk signature scores in distinct clinical stages or histology grade under (A) TCGA, (B) GSE13213, (C) GSE72094, (D) pooled cohort 1, (E) pooled cohort 2, and (F) GSE68465 datasets. * *P* < 0.05, ** *P* < 0.01, *** *P* < 0.001, NS, not significant

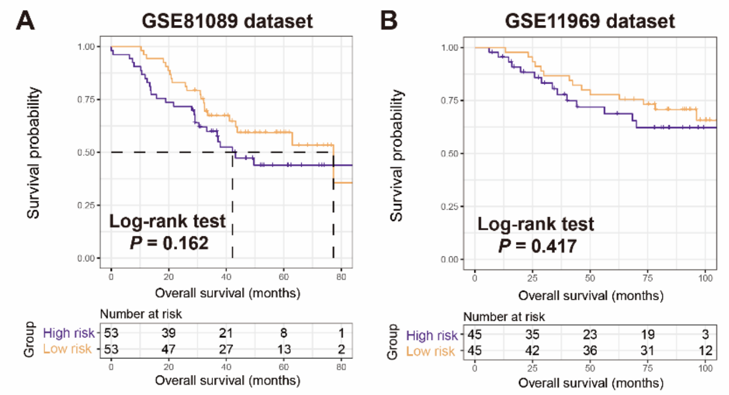

Figure S2. Kaplan-Meier survival curves stratified by LUAD patients of distinct risk levels in (A) GSE81089 and (B) GSE11969 datasets.

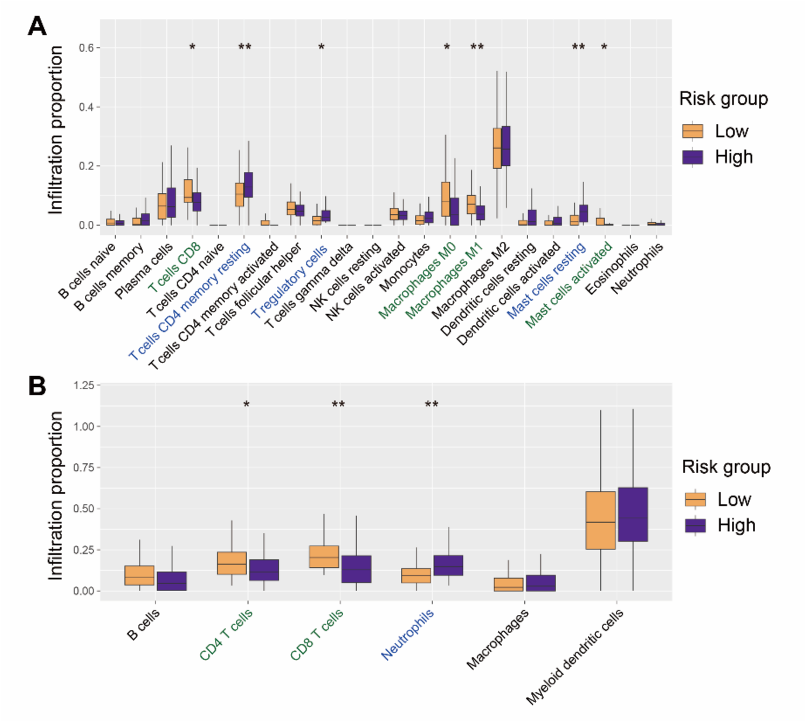

Figure S3. Distinct immunocyte infiltration abundance between low-risk and high-risk LUAD subgroups estimated by two algorithms. (A) Totaling 22 immune cell types abundance in low-risk versus high-risk patients under the CIBERSORT method. (B) Totaling 6 immune cell types abundance in low-risk versus high-risk patients under the TIMER method. Immunocytes highlighted with green were significantly enriched in low-risk patients, while those highlighted with blue were significantly enriched in high-risk patients. * $P < 0.05$, ** $P < 0.01$

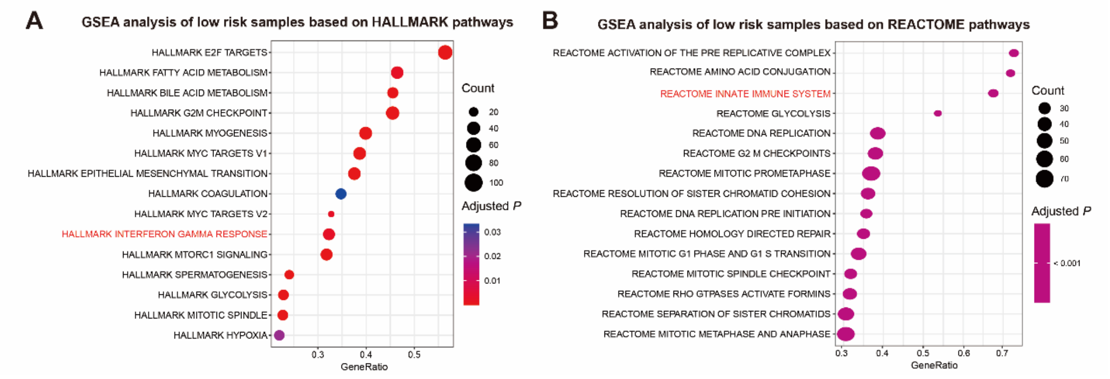

Figure S4. GSEA pathway analysis of low-risk LUAD patients based on (A) the HALLMARK database and (B) the REACTOME database. Pathways highlighted with red were immune response-related.

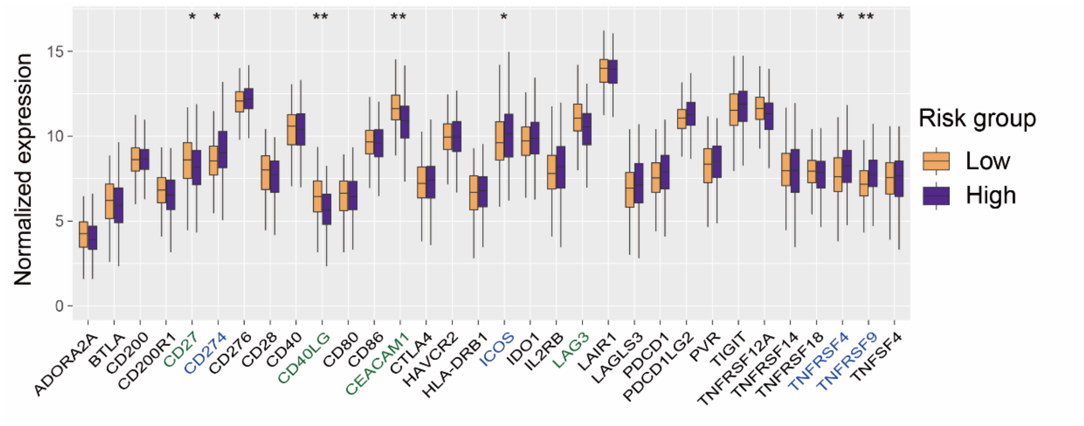

Figure S5. Distinct immune checkpoint gene expression levels in low-risk versus high-risk subgroups. Genes highlighted with green were significantly enriched in low-risk patients, while those highlighted with blue were significantly enriched in high-risk patients. * $P < 0.05$, ** $P < 0.01$

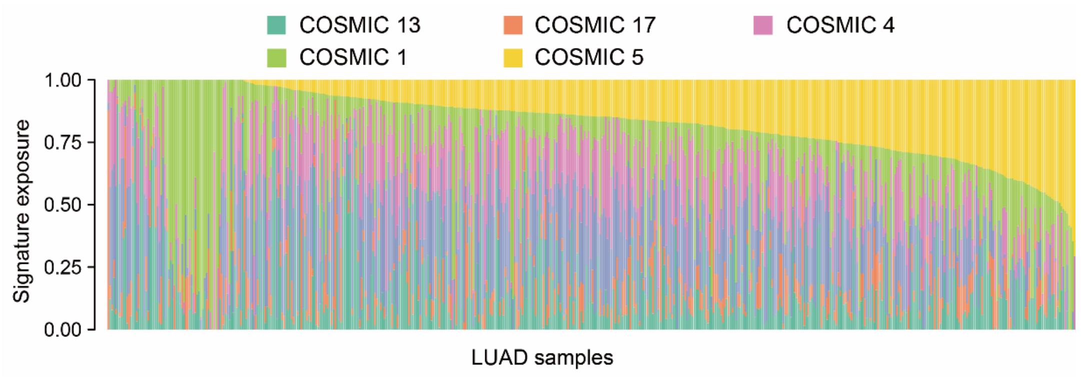

Figure S6. The distribution of mutational activities of extracted five mutational signatures across all LUAD patients in the discovery dataset.

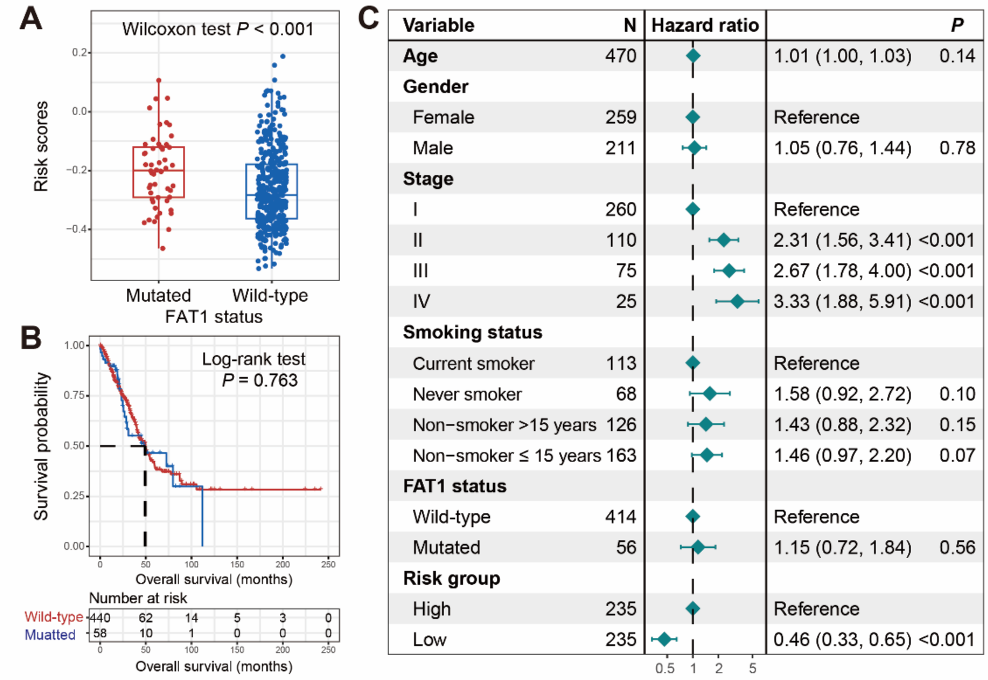

Figure S7. The distribution of risk scores in FAT1 mutated and wild-type LUAD patients and the prognostic ability of FAT1 mutations. (A) Box plot representation of signature risk scores in FAT1 two subgroups. (B) Kaplan-Meier survival analysis of FAT1 two subgroups. (C) Multivariate Cox regression model of FAT1 mutations with several clinical confounders taken into consideration.

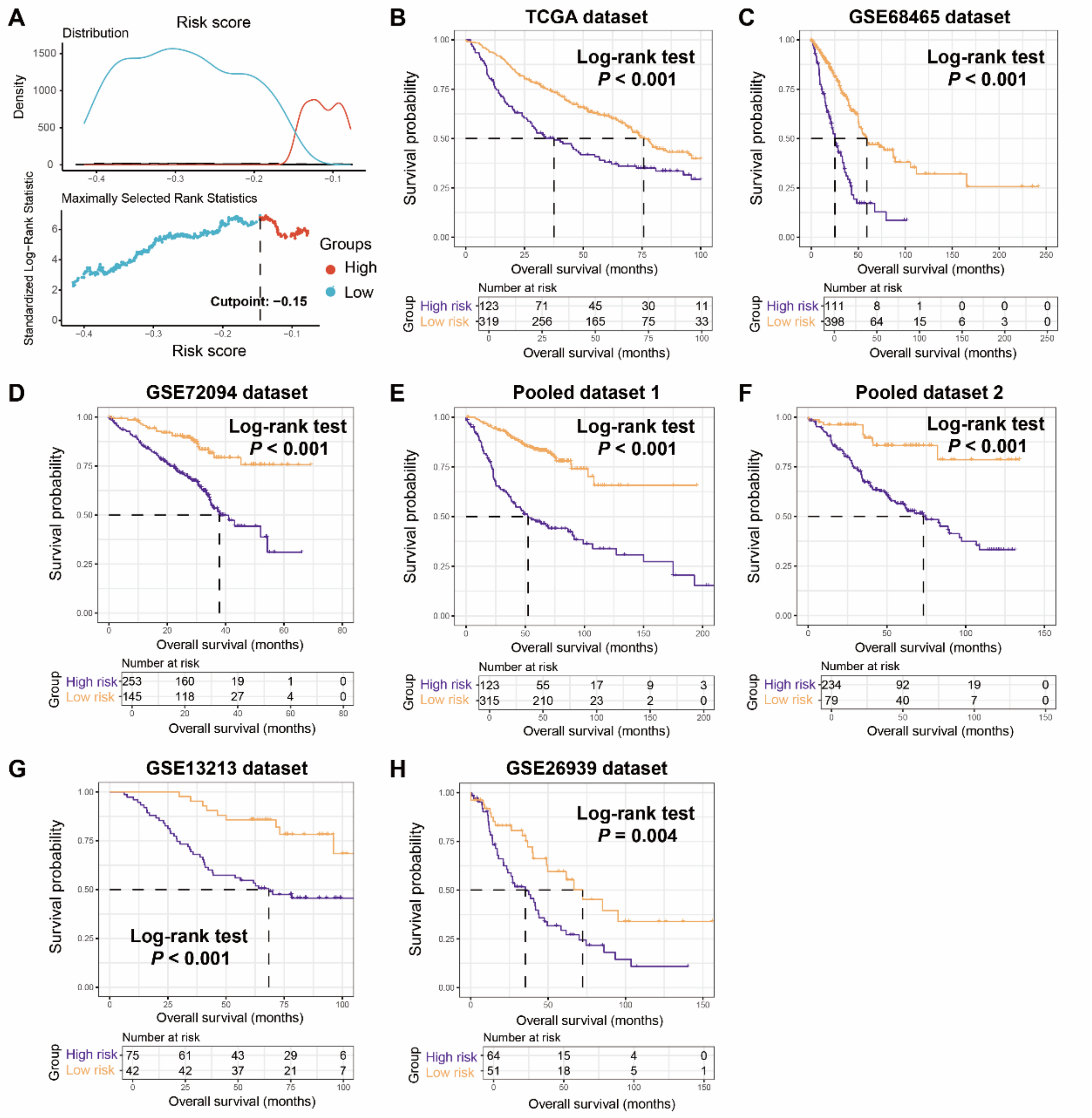

Figure S8. Kaplan-Meier survival analyses of signature risk scores stratified by cut-off values obtained from R survminer package function. (A) Schematic diagram of obtaining the risk score cut-off value based on the TCGA dataset. Kaplan-Meier survival curves of high and low-risk subgroups in (B) TCGA dataset, (C) GSE68465 dataset, (D) GSE72094 dataset, (E) pooled dataset 1, (F) pooled dataset 2, (G) GSE13213 dataset, and (H) GSE26939 dataset.

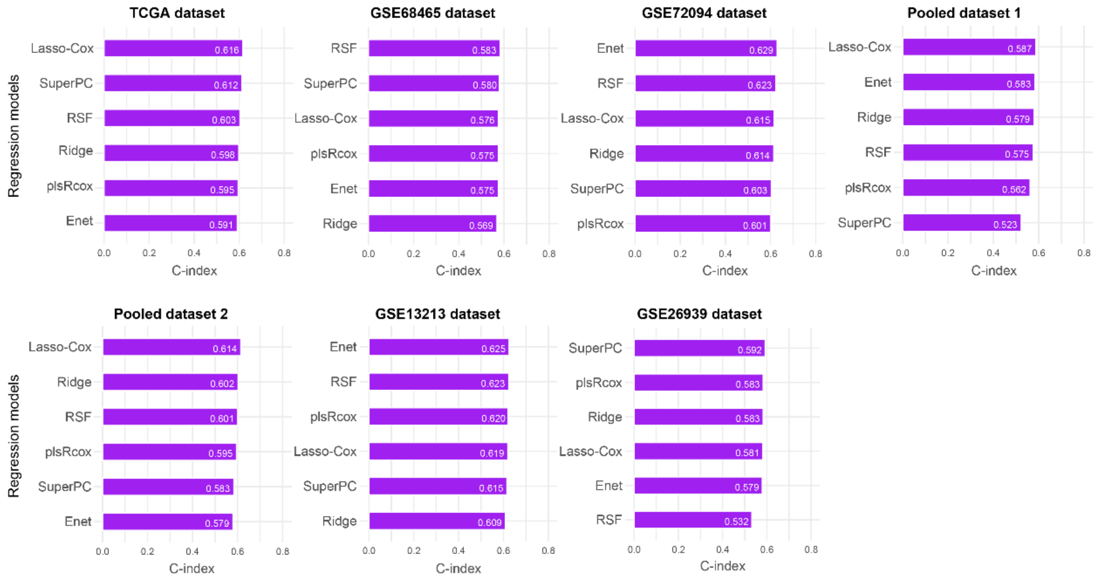

Figure S9. Presentation of the C-index for all seven LUAD cohorts included in this study under distinct prognostic models.
